# American Medical Association

**Published:** 1859-02

**Authors:** 


### AMERICAN MEDICAL ASSOCIATION.

The twelfth Annual Meeting of this Association will be held in Louisville, Kentucky, on Tuesday, May 3d, 1859. The secretaries of all societies, and other bodies entitled to representation in the Association, are requested to forward to the Secretary, S. M. Bemiss, at Louisville, correct lists of their delegations so soon as they may be appointed. The Convention of Teachers, invoked by a resolution of the National Association, for the purpose of a general conference as to the best means of elevating the standard of Medical Education in this country, will meet in the same city on Monday, the 2d of May.

Medical Journals throughout the United States are requested to insert the above.

S. M. BEMISS, M.D., *Sec. Am. Med. Assoc'n.*

By the above official notice, our readers will be warned that the next annual meeting of the American Medical Association is near at hand.

We trust that the profession of Illinois and the whole North-west will be well represented. Let every state and local society see that delegates are appointed, and their credentials furnished them in season. Louisville is easy of access, and we are sure that the local committee of arrangements will spare no pains to render the meeting both pleasant and profitable. It will be observed that on Monday preceding the day for the meeting of the Association, a Convention of Delegates from Medical Colleges is called, for adopting some means for improving the system of college instruction. One year ago, when this subject was in the hands of a special committee of the Association, we gave our views fully and freely, through the medium of this Journal. Those views remain unchanged; and any who may wish to refer to them will be able to do so, by consulting the *Chicago Medical Journal* for March, 1858. At that time we took occasion to consult many of the most eminent men connected with the medical colleges in different parts of the country, and were happy to find that their views corresponded closely with their own.

The only objections urged against adopting the proposed plan, was the want of concert among the schools, and the radical character of the changes. The first of these objections is a real obstacle in the way of any change. But we hope there may be a sufficient representation from the colleges in the proposed convention, and sufficient unity of purpose, to overcome this. The second objection should be allowed no influence whatever; simply because the errors and defects of the present system of medical college instruction are *radical* and inherent, and consequently cannot be removed without changes equally radical. Again, it would require no more concert of action and no more advertising to effect a thorough change, than it would a slight or inefficient one. The defects of our present system arise from the attempt to crowd upon the student's mind too many lectures the same day; to include the whole field of medical science in a single term of too limited a duration; and the absence of any natural order of succession in the presentation of the different branches.

No change will remedy these defects, which does not, at least, double the length of the annual college term, and divide it into two parts, with a corresponding division of the several branches, as fully set forth in the number of this Journal already alluded to. It would be better to do nothing than to adopt any mere temporizing expedients, which, when fully carried out, would still fail to remedy any of the important evils belonging to the present system.

